# Analytic performance of PANArray HPV and HPV 9G DNA chip tests for genotyping of high-risk human papillomavirus in cervical ThinPrep PreservCyt samples

**DOI:** 10.1371/journal.pone.0224483

**Published:** 2019-10-31

**Authors:** Jiyoung Kim, Sun-Young Jun

**Affiliations:** Department of Pathology, Incheon St. Mary’s Hospital, The Catholic University of Korea, Seoul, Republic of Korea; Rudjer Boskovic Institute, CROATIA

## Abstract

The detection of high-risk human papillomavirus (HR-HPV) is important for early diagnosis of precancerous cervical lesion. The distribution of HR-HPV genotypes in East Asia is different from that in Western countries. HR-HPVs non-16/18 including HPV-58 are highly prevalent in East Asia. Thus, a variety of HPV tests that could identify individual genotypes have been widely used. HPV 9G DNA is a deoxyribonucleic acid-based chip test, while PANArray HPV chip is a peptide nucleic acid-based array. We compared the analytic performance of these two chips for detecting and genotyping HR-HPV using 356 liquid-based cytology specimens and evaluated their diagnostic accuracies based on direct sequencing. For identifying HR-HPV, PANArray HPV and HPV 9G DNA chips agreed with each other for 85.1% of samples. Overall strength of agreement between the two tests was substantial (*k* = 0.68). Specifically, these two tests almost perfectly agreed for detecting several types of HR-HPV, including HPV-16, -18, -35, -52, -58, and -59 (*k*>0.81 for all). According to direct sequencing, PANArray HPV produced consistently higher sensitivities for detecting HR-HPV than HPV 9G DNA for either overall or individual genotypes of HR-HPV. Sensitivities and specificities for detecting HPV-58 were perfect (100%) with PANArray HPV. In conclusion, PANArray HPV is more effective than HPV 9G DNA in detecting HR-HPV. It is more useful for regions with high prevalent HPV-58 infection.

## Introduction

Although Papanicolaou (Pap) test contributes to incidence reduction of cervical cancer, Pap screening test alone was limited due to its low sensitivity and reproducibility for detecting high-grade squamous intraepithelial lesions (HSILs) of uterine cervix [1]. Today, liquid-based cytology has replaced conventional Pap to improve sample quality, diagnostic accuracy, and the ability to perform reflex molecular testing. In addition, human papillomavirus (HPV) co-testing with cytology has been recommended to enhance the diagnostic accuracy for screening. However, the importance of HPV detecting test as a screening tool has increased nowadays in accordance with recent approval by the US Food and Drug Administration (FDA) about the use of Cobas 4800 HPV (Roche Molecular Systems Inc., Pleasanton, CA, USA) alone as a first-line screening test in women 25 years and older [2, 3]. Among about 18 high-risk HPV (HR-HPV) genotypes identified in genital tracts, HPV-16 and HPV-18 are the most prevalent HR-HPVs of cervical cancer worldwide [4]. Cobas 4800 HPV can detect HPV-16 and HPV-18. However, it lumps 12 other HR-HPV non-16/18 types together as a pooled result [3].

The distribution of HR-HPV genotypes in East Asia is significantly different from that in Western populations [5]. HPV-58 has been highly prevalent in East Asia [5]. Ethnic genetic differences and HPV-58 variants with different oncogenicity might play a role [5]. HPV-58 contributes to only 3.3% of cervical cancers globally [5]. However, it ranks third in Asia overall [5]. In Korea, 5-year cumulative incidence rates of HSIL in women with HPV-positive and cytology-negative results were higher in HPV-58 positive cases than those in HPV-16 positive and other types of HR-HPV positive cases [6]. Therefore, HPV-16 and/or HPV-18 detection alone is insufficient for predicting the development of HSIL [5]. The identification of HPV-58 is important to manage women in a country with a high prevalent HPV-58 infection [6]. In addition, several HPV genotyping tests, mostly polymerase chain reaction (PCR)-based deoxyribonucleic acid (DNA) microarray methods, are widely used in Korea. Among HPV genotyping tests, HPV 9G DNA chip (Biometrix Technology Inc., Chuncheon, Korea) can detect 19 HPVs individually, including 14 high-risk (HR) and 5 low-risk (LR) types at the same time [7, 8](Table 1).

**Table 1:** Comparison of features of HPV chip tests.

Parameter	HPV 9G DNA	PANArray HPV
Target	L1 gene	L1 gene
Microarray platform	DNA-based	PNA-based
Detection	14 HR-HPVs and 5 LR-HPVs	19 HR-HPVs and 13 LR-HPVs
HR-HPV	HPV-16, -18, -31, -33, -35, -39, -45, -51, -52, -56, -58, -59, -66, -68	(HPV-16, -18, -31, -33, -35, -39, -45, -51, -52, -56, -58, -59, -66, -68), and HPV-26, -53, -69, -70, -73
LR-HPV	HPV-6, -11, -34, -40, -42	(HPV-6, -11, -34, -40, -42), and HPV-32, -43, -44, -54, -55, -62, -81, -83

Abbreviations: DNA, deoxyribonucleic acid; PNA, peptide nucleic acid; HR-HPV, high-risk human papillomavirus; LR-HPV, low-risk human papillomavirus.

PANArray HPV chip (Panagene Inc., Daejeon, Korea) is a peptide nucleic acid (PNA)-based test that could detect five more HR-HPV and eight more LR-HPV genotypes than HPV 9G DNA chip (Table 1)[9]. PNA is an artificial molecule having exceptional biochemical stability [9–11]. It is used as an advanced tool for many biochemical applications, including detection of DNA and alterations of gene expression and therapeutic agent-related genes [9–11]. Specific hybridization can occur between PNA and DNA due to similar intramolecular distances and configurations of nucleobases between them [9, 10].

In this study, we compared the performance of PANArray HPV and HPV 9G DNA chip tests for detecting and genotyping HR-HPVs. In addition, we validated genotyping results of these two tests by direct sequencing to compare their diagnostic accuracies.

## Materials and methods

### Patients and samples

We collected data from our electronic pathologic database between July 2012 and October 2013. A total of 1632 women who were co-tested using ThinPrep pap (Hologic Inc., Marlborough, MA, USA) and HPV 9G DNA tests for routine screening were selected. Of 1632 women, 390 (23.9%) had histological confirmative diagnosis for uterine cervix during follow-up period. They did not have previous histories for gynecologic diseases or gynecologic procedures such as colposcopic biopsy, conization, or hysterectomy. Of these 390 women, 356 with adequate remnant DNA specimen were included in our study. All DNA specimens were stored at -70°C until they were re-assayed for HPV 9G DNA and PANArray HPV tests and direct sequencing. This study was approved by the Institutional Review Board of the Catholic University of Korea, Incheon St. Mary’s Hospital (approval number: OC14EISI0081). No consent was given because data were analyzed anonymously.

### HPV chip tests

**Fig 1:**
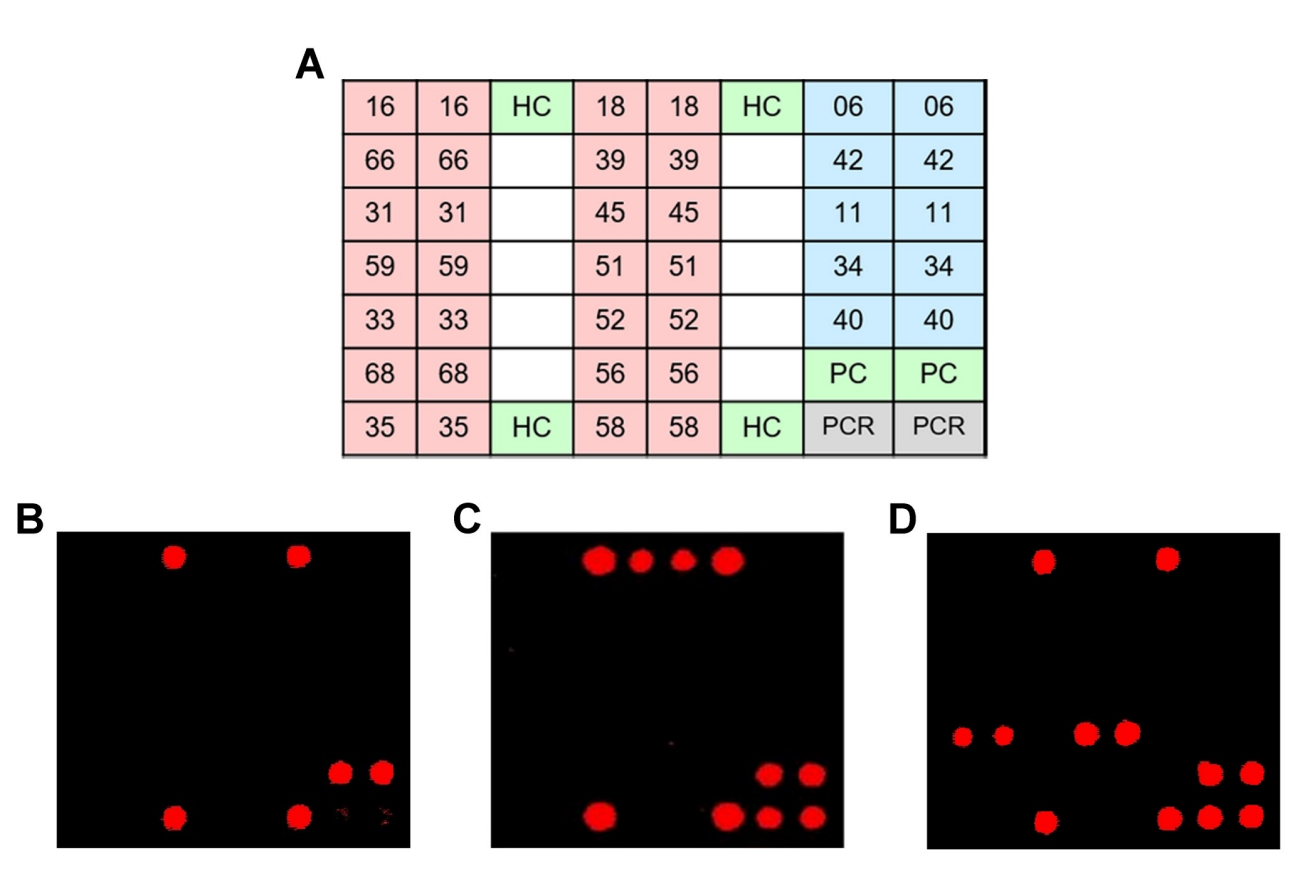
HPV 9G DNA. (a) The findings of 19 HPV genotypes including 14 high-risk group (pink color) and 5 low-risk group (blue color), along with the hybridization control (HC), PCR internal control (PC), and HPV PCR screening (PCR) probes, were spotted. Representative scanning images of (b) negative control, (c) a single infection of HPV-18, and (d) dual infections of HPV-33 and HPV-52.

HPV 9G DNA tests were performed according to the manufacturer’s protocol [7, 8, 12]. Whole HPV genomic DNA was extracted from cervical swab samples and amplified by duplex PCR to generate amplicons. After PCR amplification, 5 μL of Cy5-labeled PCR product was electrophoresed in a 2% agarose gel to confirm successful amplification by PCR. PCR product was hybridized with type-specific oligonucleotide probes and visualized on HPV 9G DNA chips as double-positive spots when HPV DNA was present in the amplified PCR product (Fig 1).

**Fig 2:**
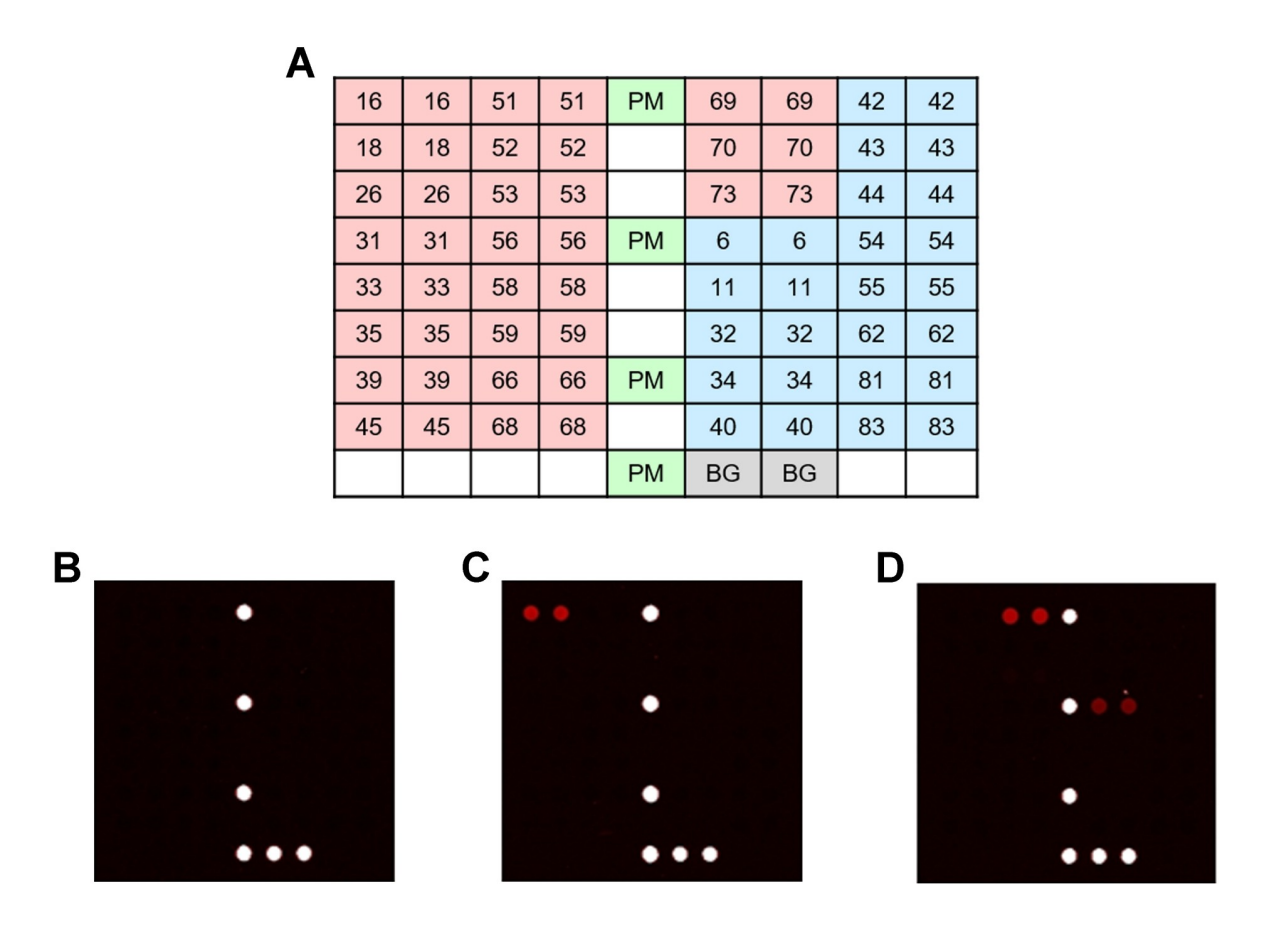
PANArray HPV. (a) The findings of 32 HPV genotypes containing 19 high-risk group (pink color) and 13 low-risk group (blue color) were spotted in duplicate with position marker (PM). ß-globin (BG) was amplified using PCR as an internal control. Representative scanning images of (b) negative control, (c) a single infection of HPV-16, and (d) double infections of HPV-6 and HPV-51.

PANArray HPV tests were performed following the manufacturer’s instructions in a similar way of HPV 9G DNA chip [9]. One PCR mix contained 5 μL of target DNA, 3 μL of PCR primer set #1 (provided by the manufacturer), and 17 μL of reaction mixture #1 containing Taq DNA polymerase, PCR buffer, and deoxynucleoside triphosphate mixture for a total volume of 25 μL. Another PCR mix contained 5 μL of the same target DNA, 3 μL of PCR primer set #2 (provided by the manufacturer), and 17μL of reaction mixture #2. After PCR amplification, each 5 μL of PCR products #1 and #2 was mixed with a mixture of hybridization buffer #1 and #2 (70 μL) and then applied to the PANArray chip. Detected HPV genotypes were presented as double-positive spots on PANArray HPV array (Fig 2). In both HPV 9G DNA and PANArray HPV tests, normal cell lines (provided by each manufacturer) were used as negative controls. None of the negative controls revealed HPV positivity. Array images of both tests were scanned using a fluorescent scanner (EasyScan-100, Xillux Co. Ltd., Seoul, Korea).

### Direct sequencing

HPV genotypes of all 356 samples were completely examined by PCR and direct sequencing. PCR was performed using general primer MY09/11 [13]. PCR products were electrophoresed and DNA of samples with a positive band was purified. In samples with discrepant results between HPV 9G DNA and PANArray HPV assays or those with multiple infections by these 2 tests, type specific primers [14] were applied again to obtain specific PCR products. We used type specific primers [14], being able to define 19 HPVs which were commonly detected by both HPV 9G DNA and PANArray HPV tests (S1 Table). Five types of HR-HPVs (including HPV-26, -53, -69, -70, and -73) and 8 LR-HPVs (including HPV-32, -43, -44, -54, -55, -62, -81, and -83) were excluded from the analysis because they could only be detected by PANArray HPV. Respective sequences of HPV DNA regions were sequenced using an Applied Biosystems 3730XL DNA analyzer (Life Technologies Co., Carlsbad, CA, USA). Specific HPV genotypes were confirmed with Basic Local Alignment Search Tool (BLAST) database on the website of the National Center for Biotechnology Information (NCBI).

### Statistical analysis

Statistical analyses were performed using Analyse—it Method Evaluation Edition software version 5.30 (Analyse-it Software Ltd., Leeds, UK). We analyzed the data according to genotyping results of 19 HPVs, which were commonly detected by both tests (S2 Table). Concordance rate, kappa coefficient (*k*) with 95% confidence intervals (CIs), proportions of positive and negative agreement, and McNemar’s *P*-values were calculated to assess agreement between HPV 9G DNA and PANArray HPV tests. The *k* value was interpreted as follows: 0–0.20, slight; 0.21–0.40, fair; 0.41–0.60, moderate; 0.61–0.80, substantial; and 0.81–1, almost perfect agreement. The proportion of positive agreement was calculated as twice the number of agreed positives/(total number of specimens + number of agreed positives–number of agreed negatives). In addition, the proportion of negative agreement was calculated as twice the number of agreed negatives/(total number of specimens—number of agreed positives + number of agreed negatives)[15]. Sensitivities, specificities, positive predictive values, and negative predictive values with 95% CIs of each test were calculated based on results of direct sequencing. When there were discrepancies among results, the diagnostic accuracy was determined by genotyping results of direct sequencing. Categorical variables were analyzed by χ^2^ or Fisher’s exact tests. Statistical significance was considered when *p* value was less than 0.05.

## Results

### Comparison between HPV 9G DNA and PANArray HPV

We compared overall frequencies of 14 HR-HPVs and 5 LR-HPVs that could be identified by both tests (Table 2).

**Table 2:** Type specific agreement for each HPV type detected by HPV chip tests.

Genotypes (*n* = 356)	Detection of HPV, *n* (%)		Agreement rate (%)	*k* (95% CI)	P*pos*	P*neg*	*P*-values
	HPV 9G DNA	PANArray HPV					
Any of 14 HR-HPVs	212 (59.6)	233 (65.4)	85.1	0.68 (0.61–0.76)	0.93	0.74	**0.005**
HR-HPVs non-16/18/58	112 (31.5)	126 (35.4)	88.2	0.74 (0.66–0.81)	0.88	0.89	**0.04**
HR-HPV							
16	64 (18.0)	66 (18.5)	96.1	0.87 (0.80–0.94)	0.91	0.97	0.87
18	14 (3.9)	15 (4.2)	98.6	0.82 (0.67–0.97)	0.86	0.99	1
58	25 (7.0)	34 (9.6)	97.5	0.83 (0.73–0.94)	1	0.97	**0.004**
31	11 (3.1)	19 (5.3)	97.8	0.72 (0.54–0.91)	1	0.98	**0.008**
33	21 (5.9)	17 (4.8)	97.8	0.78 (0.63–0.93)	0.71	0.99	0.29
35	19 (5.3)	21 (5.9)	98.3	0.84 (0.72–0.97)	0.90	0.99	0.69
39	12 (3.4)	16 (4.5)	97.8	0.70 (0.51–0.90)	0.83	0.98	0.29
45	1 (0.3)	2 (0.6)	99.7	0.67 (0.05–1.00)	1	1	1
51	12 (3.4)	22 (6.2)	96.6	0.63 (0.44–0.82)	0.92	0.97	**0.006**
52	24 (6.7)	33 (9.3)	97.5	0.83 (0.72–0.94)	1	0.97	**0.004**
56	14 (3.9)	13 (3.7)	98.0	0.73 (0.54–0.92)	0.71	0.99	1
59	1 (0.3)	1 (0.3)	100	1	1	1	1
66	11 (3.1)	14 (3.9)	98.6	0.79 (0.62–0.97)	0.91	0.99	0.38
68	14 (3.9)	18 (5.1)	96.6	0.61 (0.40–0.81)	0.71	0.98	0.39
LR-HPV							
6	2 (0.6)	2 (0.6)	100	1	1	1	1
11	1 (0.3)	1 (0.3)	100	1	1	1	1
34	2 (0.6)	1 (0.3)	99.2	0	0	1	1
40	8 (2.2)	7 (2.0)	98.6	0.66 (0.38–0.94)	0.63	0.99	1
42	6 (1.7)	6 (1.7)	98.0	0.45 (0.11–0.79)	0.43	0.99	1

Abbreviations: HPV, human papillomavirus; *k*, kappa coefficient; CI, confidence interval; P*pos*, proportion of positive agreement; P*neg*, proportion of negative agreement; HR-HPV, high-risk human papillomavirus; LR-HPV, low-risk human papillomavirus.

Bold font indicates statistically significant (*p* < 0.05).

Overall positive rates for HR-HPV were higher (233/356, 65.4%) with PANArray HPV than those with HPV 9G DNA (212/356, 59.6%), regardless of HPV genotype (Table 2). Among HR-HPVs, HPV-16, -18, and -58 were detected in more samples by PANArray HPV. Positive rates of HR-HPV genotypes non-16/18/58 were also higher with PANArray HPV.

Results of the two tests showed substantial agreement in 85.1% of all cases (*k* = 0.68) when detecting any of 14 HR-HPV types. For detecting HPV-16, results of both assays almost perfectly corresponded to each other (concordance rate, 96.1%; *k* = 0.87). Both assays also produced almost perfect agreements for HPV-18 results (concordance rate of 98.6%, *k* = 0.82) and HPV-58 results (concordance rate of 97.5%, *k* = 0.83). When considering HR-HPV genotypes of non-16/18/58, results of both HPV tests well agreed with each other at a rate of 88.2% (*k* = 0.74), which was similar to the overall concordance rate of both assays regardless of HR-HPV genotype. Statistically significant differences were seen in the detection of HPV-58, non-16/18/58 HPVs, and any of the 14 HR-HPV types (*p* = 0.004, 0.04, and 0.005, respectively). Regardless of HPV genotype, proportions of positive and negative agreement for HR-HPV status were 0.93 and 0.74, respectively. The proportion of positive agreement was higher than that of negative one for HPV-58. On the contrary, the proportion of negative agreement was higher than that of positive one for HPV-16, HPV-18, and HPVs non-16/18/58.

Type specific agreement of HR-HPVs non-16/18/58 detected by both assays are described in Table 2. The strength of agreement for detecting HPV-35 and HPV-52 was almost perfect (*k* = 0.84 and 0.83, respectively), similar to that for HPV-16, -18, and -58. For detecting HPV-59, both tests perfectly agreed (*k* = 1). For other HR-HPV genotypes including HPV-31, -33, -39, -45, -51, -56, -66, and -68, results of both tests corresponded well (0.61 ≤ *k <* 0.80 for all). Statistically significant differences were present between the two tests for the detection of HPV-31, -51, and -52 (*p* = 0.008, 0.006, and 0.004, respectively). The proportions of negative agreement for all HR-HPV non-16/18/58 types were more than 0.97 and their proportions of positive agreement were more than 0.90 except for HPV-33, -39, -56, or -68 which had a rate between 0.71 and 0.83.

Among LR-HPVs, HPV-6 and HPV-11 were detected by both assays with perfect agreement (*k* = 1, both) and HPV-40 was detected with substantial agreement (*k* = 0.66). However, these two assays moderately agreed with each other for HPV-42 results (*k* = 0.45). They did not show agreement for HPV-34. Proportions of positive and negative agreement for HPV status of LR-HPV types including HPV-6 and HPV-11 were 1. For HPV-34, -40, and -42, proportions of negative agreement for HPV status were high (over a rate of 0.99) and their proportions of positive agreement were low (from 0 to 0.63).

### Direct sequencing

Of 356 samples, HR-HPVs were identified in 232 (65.2%) by direct sequencing. Of 232 HR-HPV-positive cases, 53 (22.8%) showed multi-infection of HR-HPVs. On the other hand, LR-HPVs were seen in 19 (19/356, 5.3%) cases, 73.7% (14/19) of which were co-infected with HR-HPVs. HPV-16 was the most common type of HR-HPV (66/232, 28.5%), either alone or in combination with other types. HPV-58 was the second most common type of HR-HPV (34, 14.7%), followed by HPV-52 (32, 13.8%), HPV-35 (22, 9.5%), HPV-33 and HPV-51 (21, 9.1%, both), HPV-31 (19, 8.2%), HPV-68 (17, 7.3%), HPV-39 (16, 6.9%), HPV-18 (15, 6.5%), HPV-56 and HPV-66 (13, 5.6%, both), HPV-45 (2, 0.9%), and HPV-59 (1, 0.4%). Genotypes of LR-HPVs were infrequently observed (less than 4% in all).

### Sensitivities and specificities of HPV detection assays to identify HR genotypes

Of 356 samples, 124 without any genotype of HR-HPV confirmed by direct sequencing results were regarded as true negative. The other 232 samples were HR-HPV-positive by direct sequencing.

Based on results of direct sequencing, results of both assays for HR-HPVs were classified as concordant, compatible, and discordant: 1) concordant, when all detected HR-HPV types perfectly agreed between the two tests (true positive); 2) compatible, if agreement was made for one or more but all HR-HPV types; and 3) discordant, when there was no identical HR-HPV genotype results between the two tests (Table 3). Among 232 HR-HPV-positive samples, 161 (69.4%) were completely concordant for HR-HPV genotypes. They were considered as true positive. HR-HPV genotype results were discordant in 41 (17.7%) samples and compatible in 30 (12.9%). The frequency of concordant results was significantly higher in single infection of HR-HPV than that in multiple infections. This was consistently found in all HR-HPV genotypes (*p* < 0.05 for all, Table 3).

**Table 3:** Agreement of HPV chip tests for HR-HPV genotypes according to direct sequencing.

Genotypes	Agreement	Infections (*n*, %)			*P*-values
		Overall (*n*)	Single	Multiple	
Any of 14 HR-HPVs	Concordant	161	143 (88.8)	18 (11.2)	**< 0.001**
	Compatible	30	3 (10.0)	27 (90.0)	
	Discordant	41	33 (80.5)	8 (19.5)	
HPV-16	Concordant	50	44 (88.0)	6 (12.0)	**< 0.001**
	Compatible	10	0	10 (100)	
	Discordant	6	5 (83.3)	1 (16.7)	
HPV-18	Concordant	10	9 (90)	1 (10)	**0.03**
	Compatible	2	0	2 (100)	
	Discordant	3	2 (66.7)	1 (33.3)	
HPV-58	Concordant	22	17 (77.3)	5 (22.7)	**0.004**
	Compatible	7	1 (14.3)	6 (85.7)	
	Discordant	5	2 (40.0)	3 (60.0)	
HPVs non-16/18/58	Concordant	82	73 (89.0)	9 (11.0)	**< 0.001**
	Compatible	16	2 (12.5)	14 (87.5)	
	Discordant	27	24 (88.9)	3 (11.1)	

Abbreviations: HPV, human papillomavirus; HR-HPV, high-risk human papillomavirus.

Statistical significance (*p* < 0.05) is indicated in bold.

Sensitivities and specificities of HPV 9G DNA and PANArray HPV chip tests in terms of detected genotypes of HR-HPVs are described in Table 4. PANArray HPV showed higher sensitivity (96.1%) but lower specificity (91.6%) for the detection of any type of HR-HPVs than HPV 9G DNA (87.9% and 93.5%, respectively). For detecting HPV-16, -18, and -58 and other non-16/18/58 HR-HPVs, PANArray HPV produced consistently higher sensitivities than HPV 9G DNA (95.5% *vs*. 92.4% for HPV-16, 100% *vs*. 80.0% for HPV-18, 100% *vs*. 73.5% for HPV-58, and 94.4% *vs*. 83.2% for HPV non-16/18/58, respectively). The specificity was higher with PANArray HPV for the detection of HPV-18 (100% *vs*. 99.4%). For other HR-HPVs, the two tests showed same specificities (99.0% for HPV-16, 100% for HPV-58, and 96.5% for HR-HPVs non-16/18/58).

**Table 4:** Sensitivities and specificities of HPV chip tests for HR-HPV genotypes.

Test (%)	Sensitivity (95% CI)	Specificity (95% CI)	PPV (95% CI)	NPV (95% CI)
HPV 9G DNA				
Any of 14 HR-HPV	87.9 (83.1–91.5)	93.5 (87.8–96.7)	96.2 (92.9–98.0)	80.6 (74.5–85.5)
HPV-16	92.4 (83.5–96.7)	99.0 (97.0–99.6)	95.3 (86.8–98.4)	98.3 (96.1–99.3)
HPV-18	80.0 (54.8–93.0)	99.4 (97.9–99.8)	85.7 (59.6–96.1)	99.1 (97.6–99.7)
HPV-58	73.5 (56.9–85.4)	100 (98.8–100)	100	97.3 (95.3–98.4)
HPVs non-16/18/58	83.2 (75.7–88.7)	96.5 (93.3–98.2)	92.9 (86.8–96.3)	91.4 (87.8–94.0)
PANArray HPV				
Any of 14 HR-HPVs	96.1 (92.8–97.9)	91.6 (85.8–95.6)	95.7 (92.5–97.6)	92.7 (86.9–96.0)
HPV-16	95.5 (87.5–98.4)	99.0 (97.0–99.6)	95.5 (87.2–98.5)	99.0 (96.9–99.7)
HPV-18	100 (79.6–100)	100 (98.9–100)	100	100
HPV-58	100 (89.8–100)	100 (98.8–100)	100	100
HPVs non-16/18/58	94.4 (88.9–97.3)	96.5 (93.3–98.2)	93.7 (88.2–96.7)	97.0 (93.9–98.5)

Abbreviations: HPV, human papillomavirus; HR-HPV, high-risk human papillomavirus; CI, confidence interval; PPV, positive predictive value; NPV, negative predictive value.

## Discussion

We compared results of HPV 9G DNA and PANArray HPV chip tests for 356 cervical ThinPrep PreservCyt samples and analyzed their diagnostic accuracies with reference standard of direct sequencing results. Although these two tests had similar techniques of target amplification and hybridization, PANArray HPV chip was superior to assay HR-HPVs than HPV 9G DNA with higher sensitivity and specificity. In particular, PANArray HPV perfectly detected HPV-58. Thus, this method may be more useful in countries with high prevalence of HPV-58 infection.

Although non-16/18 HR-HPV genotypes also play a significant role in cervical neoplasia, current screening and management algorithms separate out these HPV types from HPV-16 and HPV-18 [16]. HPV-16 and HPV-18 have been classified as cervical carcinogens for a long time since 1995. Other HR-HPV non-16/18 were concerned early in 2000 [17]. The overall prevalence of non-16/18 HR-HPV genotypes varied with populations and regions studied. In Korea, HPV-58 was revealed as the second most commonly detected type by HPV tests following HPV-16 [18]. On the other hand, in a recent study in China, HPV-52 was the most common HR genotype followed by HPV-16, -58, -39, -18, and -56 [19]. Even in the United States, the large Addressing the Need for Advanced HPV Diagnostics (ATHENA) trial of women (≥ 25 years) with HPV co-testing found that HPV-52 was the second most prevalent HR genotype [20]. Therefore, attention to HR-HPV genotypes non-16/18 does not simply belong to Eastern populations. In addition, among three different HPV vaccines approved by the FDA, namely Gardasil (Merck Co. Inc., Kenilworth, NJ, USA), Gardasil 9 (Merck Co. Inc.), and Cervarix (GlaxoSmithKline Biologicals, Brentford, UK), only Gardasil 9 can cover the most significant HPV non-16/18 types including HPV-31, -33, -45, -52, and -58 [16]. Therefore, as the importance of HR-HPV genotypes non-16/18 increases, studies for epidemiologic and clinical significance of HR genotypes non-16/18 should be preceded by those to find more effective methods to detect HR-HPV genotypes non-16/18. Jun et al. [15] have revealed that Cobas 4800 HPV and HPV 9G DNA chip, both of which are DNA-based tests, have similar advantage in identifying HPV-16 and HPV-18. However, HPV 9G DNA chip is more useful to identify HR-HPV genotypes non-16/18 than Cobas 4800 HPV [15]. PANArray HPV was developed in Korea in 2009 [9]. Although a few previous studies have compared the clinical efficacy of PANArray HPV to other HPV detecting assays, the evaluation was performed with respect to the relationship with histologic and cytological diagnoses [14, 21]. However, the authors did not compare diagnostic accuracies of the tests for assaying this specific HPV genotype [14, 21]. Therefore, we focused on highly prevalent HR-HPV genotypes worldwide (HPV-16 and HPV-18) and in East Asia (HPV-58) and purely compared analytic performance of HPV tests using DNA or PNA using result of direct sequencing as reference. In the present study, we revealed unprecedented sensitivity and specificity for detecting and genotyping all HR-HPV with PNA-based HPV array, including HPV-58. This is probably contributed to the more accurate PNA probes that could make greater stability of PNA-DNA hybrids than their DNA-DNA counterparts. Recently, PANA RealTyper HPV (Panagene Inc.), an amplified DNA test for the qualitative detection of HPV genotypes using PNA proves and melting temperature in a real-time PCR system, is used in Korea. Effectiveness of these PNA-based HPV tests for identifying HPV genotypes between chips and real-time PCR needs to be compared and evaluated.

In a monograph from International Agency for Research on Cancer (IARC), 12 HPV types (HPV-16, -18, -31, -33, -35, -39, -45, -51, -52, -56, -58, and -59) were classified as “carcinogenic to humans (Group 1)” and another 8 types (HPV-26, -53, -66, -67, -68, -70, -73, and -82) were classified as “probably or possible carcinogenic to humans (Group 2)” [17]. PANArray could identify five more genotypes of HR-HPVs mostly within the group 2 than HPV 9G DNA. Of 356 samples, we found HPV-53 in 6.5% (23 samples), HPV-70 in 4.5% (16), HPV-69 in 3.4% (12), and HPV-26 in 0.6% (2), resulting in 15.0% overall. There was no HPV-73 identified in this study. The number of samples used for detecting these HR-HPV genotypes was not negligible. Further studies are needed to define their clinical significance.

In conclusion, PANArray HPV had higher sensitivity and specificity in detecting HR-HPV than HPV 9G DNA. In particular, PANArray HPV perfectly detected HPV-58. Therefore, PANArray HPV is more effective than HPV 9G DNA for detecting HR-HPV. It is considered to be more useful for regions with high prevalence of HPV-58 infection, including Korea.

## Supporting information

S1 Table Sequences of type specific primers. (DOCX)

S2 Table HPV genotyping results detected by HPV 9G DNA and PANArray HPV tests and direct sequencing. (DOCX)
