# Supplementary material for: Analytic performance of PANArray HPV and HPV 9G DNA chip tests for genotyping of high-risk human papillomavirus in cervical ThinPrep PreservCyt samples
Source: PLoS One. 2019 Oct 31;14(10):e0224483. doi: 10.1371/journal.pone.0224483 (PMC6822940; doi:10.1371/journal.pone.0224483)
Supplement: S1 Table — (DOCX) [file pone.0224483.s001.docx]

| **Type** | **Accession No.** | **Primer** | **Sequence (5’-3’)** |
| --- | --- | --- | --- |
| HPV-6 | HG793922 | 6F | CAC CTA AAG GTC CTG TTT CGA GGC GGC TAT |
|  |  | 6R | CTG AAT CGT CCG CCA TCG TTG TTA GGT CTT |
| HPV-11 | KC329894 | 11F | TGG AGT GCA CAG ACG GAG ACA TCA GAC AAC |
|  |  | 11R | TTA AAC AAT GCC TGT GCT TCC ACA GAA TTT |
| HPV-34 | X74476 | 34F | GGG TAT GTC AAC CGT GTT TAC TGT TTT ACT |
|  |  | 34R | ATT ATC AAT AAA ATC CCC CAT TTC AGA ATC |
| HPV-40 | HE793074 | 40F | AGG GCC ATA ACA ATG GCA TA |
|  |  | 40R | TGG AAA TTG ATC TAA TTG GGA AG |
| HPV-42 | GQ472847 | 42F | CAG CTA AAC GTA AGA AAA CAC ACA AAT AGA |
|  |  | 42R | CTT ATT TTT CAA AGC CAG GAT TGT AGT TTA |
| HPV-16 | KP874716 | 16F | TTT ATA CAT TAA AGG CTC TGG GTC TAC TGC |
|  |  | 16R | TAA GGT TTA TTG AAT ATT TGG GCA TCA GAG |
| HPV-18 | KT070102 | 18F | CAG TCT CCT GTA CCT GGG CAA TAT GAT GCT |
|  |  | 18R | CTA TGG TGG GCT TGC GAC GCA ATC CAG CCT |
| HPV-31 | KF700156 | 31F | TGC AAA GGT CAG TTA ACA GAA ACA GAG GTA |
|  |  | 31R | ACA GCT CTT GCA ATA TGC GAAA TAT CTA CTT |
| HPV-33 | KF700164 | 33F | AAC TAT ACA CAA CAT TGA ACT ACA GTG CGT |
|  |  | 33R | ATC TAA AAC ATA TTC CTT TAA CGT TGG CTT |
| HPV-35 | JX129488 | 35F | AGA AGT GGA CAG ACA TTG TAA GGT GCG GTA T |
|  |  | 35R | GTC ATC TTC ATT TTC GTC CTC TAC ACT GGA |
| HPV-39 | KC470245 | 39F | AAG TAT GTA TGA CAG TTT CAT GTG TGA TTG |
|  |  | 39R | ACA AAA TGG CGA AGT ATA AAA TGT AGA AAC |
| HPV-45 | KC470255 | 45F | ATT GTA TAA TTG GCG TGT AGA ACC ACT TTC |
|  |  | 45R | TTT GCA ATA TAC ACA GGC AAT AGA TAC GTC |
| HPV-51 | M62877 | 51F | TTC GGT TCG TGT ACT TTT AGT ATA TTT GCC |
|  |  | 51R | TTA AAT TAT TAT AGG GCG GAA AAC AGT GTG |
| HPV-52 | KF700237 | 52F | ATA CAG TTG CTC CTA ATC TAT TGC ATC TCC |
|  |  | 52R | GCA GGA CCT GTG AGT CAG CAA GAA GTC AGT |
| HPV-56 | JX912947 | 56F | AGA AGC ACA GCT ATA ACA TGT CAA CGG GAA C |
|  |  | 56R | CTT ACA AAA CAA AAG CCA CAA TAA TGA CAC |
| HPV-58 | AB819279 | 58F | CAG ACT AAA ACG TTC GGC CCC TAC TAC CCG |
|  |  | 58R | GGA GGT AAA GTA AAA TGG AGGG CAG TAC TGT |
| HPV-59 | KC470266 | 59F | AAA CTA CTG TGC AAT CCA AGA ATG TGT CTA |
|  |  | 59R | ATA TCA TGC AGA GGA ATA TTC AAT GTT GTG |
| HPV-66 | JN122292 | 66F | TTG ATT GTA AAC AAA CCC AGT TAT GTA TTG |
|  |  | 66R | GGG CAT CAT ATT TAG TTA ATG TGC TTT TAG |
| HPV-68 | KC70283 | 68F | TTG TAT ATT AAG GGC ACT GAC ATA CGT GAC |
|  |  | 68R | TAC AAC CAC ACA TAC AAC CAA CAT ACA AAA |
