# Supplementary material for: Analytic performance of PANArray HPV and HPV 9G DNA chip tests for genotyping of high-risk human papillomavirus in cervical ThinPrep PreservCyt samples
Source: PLoS One. 2019 Oct 31;14(10):e0224483. doi: 10.1371/journal.pone.0224483 (PMC6822940; doi:10.1371/journal.pone.0224483)
Supplement: S2 Table — (DOCX) [file pone.0224483.s002.docx]

| **No.** | **HPV genotyping results** | | |
| --- | --- | --- | --- |
|  | **HPV 9G DNA** | **PANArray HPV** | **Sequencing** |
| 1 | 56 | 56,45,70 | 56,45 |
| 2 | 16,58 | 16,58 | 16,58 |
| 3 | 33 | 31 | 31,33 |
| 4 | 33,39 | 33,39,81 | 33,39 |
| 5 | 33 | 33 | 33 |
| 6 | 52 | 52,62 | 52 |
| 7 | 18 | 18,70 | 18 |
| 8 | 16 | 16,52 | 16,52 |
| 9 | 52 | 52 | 52 |
| 10 | 58 | 58 | 58 |
| 11 | 16 | 16 | 16 |
| 12 | 52 | 52 | 52 |
| 13 | 58 | 58 | 58 |
| 14 | 33 | 33 | 33 |
| 15 | 39 | 39,31,42 | 39,31,42 |
| 16 | 16 | 16 | 16 |
| 17 | 18,34 | 18 | 18,34 |
| 18 | 16 | 16 | 16 |
| 19 | 31 | 31 | 31 |
| 20 | 16,52 | 16,52 | 16,52 |
| 21 | 16,58 | 16,58 | 16,58 |
| 22 | 33 | 33 | 33 |
| 23 | 35 | 35,53,54 | 35 |
| 24 | 35 | 35 | 35 |
| 25 | 52 | 52 | 52 |
| 26 | 35 | 35 | 35 |
| 27 | Negative | 69,35 | 35 |
| 28 | 16 | 16 | 16 |
| 29 | 35 | 35,68 | 35 |
| 30 | 51 | 51 | 51 |
| 31 | 16 | 16 | 16 |
| 32 | 35 | 35,66,53 | 35,66 |
| 33 | 52 | 52 | 52 |
| 34 | 33 | 33 | 33 |
| 35 | 16 | 16 | 16 |
| 36 | 58 | 58 | 58 |
| 37 | 52,68 | 52,68 | 52,68 |
| 38 | 52 | 52 | 52 |
| 39 | 51 | 51 | 51 |
| 40 | 16 | 16 | 16 |
| 41 | 58 | 58,31 | 58,31 |
| 42 | 16 | 16 | 16 |
| 43 | 58 | 58 | 58 |
| 44 | 16,58 | 16,58 | 16,58 |
| 45 | 31 | 31 | 31 |
| 46 | 33,51 | 33,51 | 33,51 |
| 47 | 16 | 16 | 16 |
| 48 | 18 | 32,56 | 56 |
| 49 | 16 | 16,39 | 16,39 |
| 50 | 16 | 16 | 16 |
| 51 | 16 | 16,53 | 16 |
| 52 | 33 | 33,34 | 33,34 |
| 53 | 16 | 16 | 16 |
| 54 | 16 | 16 | 16 |
| 55 | 16 | 32,53,62 | Negative |
| 56 | 52 | 52 | 52 |
| 57 | 16 | 16 | 16 |
| 58 | 16 | 16 | 16 |
| 59 | 16 | 16,53 | 16 |
| 60 | 16 | 16,53 | 16 |
| 61 | 68 | 68,16 | 68,16 |
| 62 | 16 | 16 | 16 |
| 63 | 33 | 33,51 | 33,51 |
| 64 | 33,51,42 | 33,51 | 33,51 |
| 65 | 31 | 31 | 31 |
| 66 | 31 | 31,68 | 31,68 |
| 67 | 35 | 35 | 35 |
| 68 | 35 | 35 | 35 |
| 69 | 18 | 18 | 18 |
| 70 | 18 | 18,40 | 18,40 |
| 71 | 16 | 16,43 | 16 |
| 72 | 16 | 16 | 16 |
| 73 | 35 | 35 | 35 |
| 74 | 35,56 | 56 | 35,56 |
| 75 | 18 | 18,35 | 18,35 |
| 76 | 16 | 16,81 | 16 |
| 77 | 39,56 | 39,56 | 39,56 |
| 78 | 52 | 52 | 52 |
| 79 | 35,45 | 35,45,81 | 35,45 |
| 80 | 56 | 56 | 56 |
| 81 | 68 | 68 | 68 |
| 82 | 68 | 54,56,58,53 | 68,56,58 |
| 83 | 56 | 56,16 | 56,16 |
| 84 | 52 | 52 | 52 |
| 85 | 66 | 66,52 | 66,52 |
| 86 | 39 | 39 | 39 |
| 87 | 58 | 58 | 58 |
| 88 | 52 | 52 | 52 |
| 89 | 31 | 31,62 | 31 |
| 90 | 31,35,40 | 31,62 | 31,35,40 |
| 91 | Negative | 70 | Negative |
| 92 | 68 | 68 | 68 |
| 93 | 56 | 56 | 56 |
| 94 | Negative | 70 | Negative |
| 95 | 16 | 16 | 16 |
| 96 | 16 | 16 | 16 |
| 97 | 16 | Negative | 16 |
| 98 | 33 | Negative | 33 |
| 99 | 33,59 | 33,59,68 | 33,59,68 |
| 100 | 66,68 | 66,68 | 66,68 |
| 101 | 68 | 68,53,62 | 68 |
| 102 | 66 | 83 | 66 |
| 103 | 39 | 39 | 39 |
| 104 | 51 | 51 | 51 |
| 105 | 16 | 16 | 16 |
| 106 | 16,33 | Negative | 16,33 |
| 107 | 52 | 52,53 | 52 |
| 108 | 58 | 58 | 58 |
| 109 | 52 | 52 | 52 |
| 110 | 16 | 16,58 | 16,58 |
| 111 | 16 | 16,70 | 16 |
| 112 | 16,42 | 16 | 16,42 |
| 113 | 39 | 39 | 39 |
| 114 | 16 | 16,68 | 16,68 |
| 115 | 56 | 56,42 | 56,42 |
| 116 | 16 | Negative | 16 |
| 117 | 66 | 66 | 66 |
| 118 | 58 | 58 | 58 |
| 119 | 58 | 58 | 58 |
| 120 | 16,40,42 | 16,40 | 16,40 |
| 121 | 16 | 16 | 16 |
| 122 | 39 | Negative | 39 |
| 123 | 58 | 58 | 58 |
| 124 | 16 | 16,51 | 16,51 |
| 125 | 39 | 39,81 | 39 |
| 126 | 56 | Negative | 56 |
| 127 | 68 | 69 | Negative |
| 128 | 51 | 51 | 51 |
| 129 | 18 | 18 | 18 |
| 130 | 16,33,66 | 16,33,66 | 16,33,66 |
| 131 | 51 | 51 | 51 |
| 132 | 18 | 18 | 18 |
| 133 | 52 | 52 | 52 |
| 134 | 56 | Negative | Negative |
| 135 | 52 | 52 | 52 |
| 136 | 68 | 68,31,53 | 68,31 |
| 137 | 39,56 | 39,43,54 | 39 |
| 138 | 31 | 31 | 31 |
| 139 | 58,33 | 58 | 58 |
| 140 | 51 | 51 | 51 |
| 141 | 35 | 35 | 35 |
| 142 | Negative | 70 | Negative |
| 143 | 68 | 68 | 68 |
| 144 | 35,68 | 35,53 | 35,68 |
| 145 | 66 | 66 | 66 |
| 146 | 58 | 58 | 58 |
| 147 | 33,66 | 33,66 | 33,66 |
| 148 | 39 | 39 | 39 |
| 149 | 39 | 39 | 39 |
| 150 | 56 | 56 | 56 |
| 151 | 68 | 68 | 68 |
| 152 | 58 | 58 | 58 |
| 153 | 52 | 52 | 52 |
| 154 | 18,51 | 18,51 | 18,51 |
| 155 | 51 | 58,66 | 58,66 |
| 156 | 18,31,58,56 | 18,31,58 | 18,31,58 |
| 157 | 58 | 58 | 58 |
| 158 | 33 | 33 | 33 |
| 159 | 35 | 35 | 35 |
| 160 | 18 | 18 | 18 |
| 161 | 52 | 52 | 52 |
| 162 | 16 | 16 | 16 |
| 163 | 58 | 58 | 58 |
| 164 | 16 | 16 | 16 |
| 165 | 51,66 | 51,66 | 51,66 |
| 166 | 16,35 | 16,35 | 16,35 |
| 167 | 51 | 51 | 51 |
| 168 | 56 | 56 | 56 |
| 169 | 35 | 35 | 35 |
| 170 | 52 | 52 | 52 |
| 171 | 58 | 58 | 58 |
| 172 | 56 | 56 | 56 |
| 173 | 52,58 | 52,58 | 52,58 |
| 174 | 35 | 35 | 35 |
| 175 | 16 | 16 | 16 |
| 176 | 16 | 16 | 16 |
| 177 | 33 | 33,54 | 33 |
| 178 | 16 | 16 | 16 |
| 179 | 16 | 16 | 16 |
| 180 | 58 | 58 | 58 |
| 181 | 66 | 66,58 | 66,58 |
| 182 | 35 | 35 | 35 |
| 183 | 16 | 16,52 | 16,52 |
| 184 | 16 | 16,53,70 | 16 |
| 185 | 35 | 35 | 35 |
| 186 | 16 | 16 | 16 |
| 187 | 31 | 31 | 31 |
| 188 | 66 | 66,53,69 | 66 |
| 189 | 33 | Negative | 33 |
| 190 | 52 | 52 | 52 |
| 191 | 52,34 | 52 | 52 |
| 192 | 31 | 31,58 | 31,58 |
| 193 | 16 | 16 | 16 |
| 194 | 16 | 16 | 16 |
| 195 | 16 | 16 | 16 |
| 196 | 16 | 16,69 | 16 |
| 197 | 16 | 16 | 16 |
| 198 | 16 | 16 | 16 |
| 199 | 18 | 18 | 18 |
| 200 | 18 | 18 | 18 |
| 201 | 16 | 16,58 | 16,58 |
| 202 | Negative | 70 | Negative |
| 203 | 52 | 51,52 | 51,52 |
| 204 | 31,58,68 | 31,58,68,62 | 31,58,68 |
| 205 | 66 | 66 | 66 |
| 206 | 33,42 | 33,42 | 33,42 |
| 207 | 39,6 | 6 | 39,6 |
| 208 | 18 | Negative | Negative |
| 209 | 68 | 69 | Negative |
| 210 | 16 | Negative | Negative |
| 211 | 16 | 16 | 16 |
| 212 | 16,33 | Negative | Negative |
| 213 | 58 | 58 | 58 |
| 214 | 16 | 16 | 16 |
| 215 | 16 | 16,31 | 16,31 |
| 216 | 58 | 58 | 58 |
| 217 | 40 | 33 | 33,40 |
| 218 | Negative | 43,58 | 58 |
| 219 | 52 | 52 | 52 |
| 220 | 11 | 11,16,35 | 16,11 |
| 221 | Negative | 43 | Negative |
| 222 | 40 | 40 | 40 |
| 223 | 40 | 40,54 | 40 |
| 224 | 42 | 42 | 42 |
| 225 | 40 | 40,51 | 51,40 |
| 226 | 6 | 6 | 6 |
| 227 | 40 | Negative | Negative |
| 228 | 42 | Negative | Negative |
| 229 | Negative | 33 | 33 |
| 230 | Negative | 16 | 16 |
| 231 | Negative | Negative | Negative |
| 232 | Negative | Negative | Negative |
| 233 | Negative | 16,54,58 | 58 |
| 234 | Negative | Negative | Negative |
| 235 | Negative | 51 | 51 |
| 236 | Negative | 39,53 | 39 |
| 237 | Negative | Negative | Negative |
| 238 | Negative | 81 | Negative |
| 239 | Negative | Negative | Negative |
| 240 | Negative | Negative | Negative |
| 241 | Negative | Negative | Negative |
| 242 | Negative | 43 | Negative |
| 243 | Negative | 31 | 31 |
| 244 | Negative | 68,26,53 | Negative |
| 245 | Negative | Negative | Negative |
| 246 | Negative | 44 | Negative |
| 247 | Negative | 53,69 | Negative |
| 248 | Negative | 54 | Negative |
| 249 | Negative | Negative | Negative |
| 250 | Negative | 16 | 16 |
| 251 | Negative | 16 | Negative |
| 252 | Negative | 52 | 52 |
| 253 | Negative | 51,69,70 | 51 |
| 254 | Negative | 18,51,52,69 | 18,51,52 |
| 255 | Negative | 32,53 | Negative |
| 256 | Negative | 53,81 | Negative |
| 257 | Negative | 70 | Negative |
| 258 | Negative | 44 | Negative |
| 259 | Negative | 52 | 52 |
| 260 | Negative | 44 | Negative |
| 261 | Negative | Negative | Negative |
| 262 | Negative | Negative | Negative |
| 263 | Negative | 69,70 | Negative |
| 264 | Negative | 68 | 68 |
| 265 | Negative | 53 | Negative |
| 266 | Negative | Negative | Negative |
| 267 | Negative | 26 | Negative |
| 268 | Negative | Negative | Negative |
| 269 | Negative | Negative | Negative |
| 270 | Negative | 39,53 | Negative |
| 271 | Negative | Negative | Negative |
| 272 | Negative | 66 | Negative |
| 273 | Negative | Negative | Negative |
| 274 | Negative | Negative | Negative |
| 275 | Negative | 68,39 | 68,39 |
| 276 | Negative | 70 | Negative |
| 277 | Negative | 70 | Negative |
| 278 | Negative | Negative | Negative |
| 279 | Negative | 52 | Negative |
| 280 | Negative | Negative | Negative |
| 281 | Negative | Negative | Negative |
| 282 | Negative | Negative | Negative |
| 283 | Negative | Negative | Negative |
| 284 | Negative | 31 | 31 |
| 285 | Negative | Negative | Negative |
| 286 | Negative | Negative | Negative |
| 287 | Negative | 62 | Negative |
| 288 | Negative | 18,56 | 18 |
| 289 | Negative | 51 | 51 |
| 290 | Negative | Negative | Negative |
| 291 | Negative | 51,42,40,69,70 | 51,40 |
| 292 | 40 | 40,42,69,70 | 40,42 |
| 293 | Negative | 51 | 51 |
| 294 | Negative | Negative | Negative |
| 295 | Negative | Negative | Negative |
| 296 | Negative | 53 | Negative |
| 297 | Negative | 52,58 | 52,58 |
| 298 | Negative | 66 | Negative |
| 299 | Negative | 81 | Negative |
| 300 | Negative | Negative | Negative |
| 301 | Negative | Negative | Negative |
| 302 | Negative | 35 | 35 |
| 303 | Negative | Negative | Negative |
| 304 | Negative | Negative | Negative |
| 305 | Negative | Negative | Negative |
| 306 | Negative | Negative | Negative |
| 307 | Negative | Negative | Negative |
| 308 | Negative | 31,39 | 31,39 |
| 309 | Negative | 52 | 52 |
| 310 | Negative | Negative | Negative |
| 311 | Negative | Negative | Negative |
| 312 | Negative | Negative | Negative |
| 313 | Negative | 69 | Negative |
| 314 | Negative | Negative | Negative |
| 315 | Negative | Negative | Negative |
| 316 | Negative | Negative | Negative |
| 317 | Negative | 81 | Negative |
| 318 | Negative | Negative | Negative |
| 319 | Negative | 18,53 | 18 |
| 320 | Negative | Negative | Negative |
| 321 | Negative | 62 | Negative |
| 322 | Negative | Negative | Negative |
| 323 | Negative | Negative | Negative |
| 324 | Negative | Negative | Negative |
| 325 | Negative | Negative | Negative |
| 326 | Negative | Negative | Negative |
| 327 | Negative | Negative | Negative |
| 328 | Negative | Negative | Negative |
| 329 | Negative | Negative | Negative |
| 330 | Negative | Negative | Negative |
| 331 | Negative | Negative | Negative |
| 332 | Negative | Negative | Negative |
| 333 | Negative | Negative | Negative |
| 334 | Negative | Negative | Negative |
| 335 | Negative | Negative | Negative |
| 336 | Negative | 68 | Negative |
| 337 | Negative | Negative | Negative |
| 338 | Negative | 81 | Negative |
| 339 | Negative | Negative | Negative |
| 340 | Negative | 51 | Negative |
| 341 | Negative | Negative | Negative |
| 342 | Negative | Negative | Negative |
| 343 | Negative | Negative | Negative |
| 344 | Negative | Negative | Negative |
| 345 | Negative | Negative | Negative |
| 346 | Negative | Negative | Negative |
| 347 | Negative | Negative | Negative |
| 348 | Negative | Negative | Negative |
| 349 | Negative | Negative | Negative |
| 350 | Negative | Negative | Negative |
| 351 | Negative | Negative | Negative |
| 352 | Negative | Negative | Negative |
| 353 | Negative | Negative | Negative |
| 354 | Negative | 16,39,54,70 | Negative |
| 355 | Negative | 53 | Negative |
| 356 | Negative | Negative | Negative |
